# The complexities of trans women’s access to healthcare in South Africa: moving health systems beyond the gender binary towards gender equity

**DOI:** 10.1186/s12939-023-02039-6

**Published:** 2023-11-03

**Authors:** Siyanda B. Shabalala, Megan M. Campbell

**Affiliations:** Rhodes University, Makhanda, South Africa

**Keywords:** Trans women, Medical genderism, Cisnormativity, Trans inclusive healthcare, Gender equity, Public health systems, South Africa

## Abstract

**Background:**

Public health research highlights the influence of socio-political biases shaping obstacles to fair healthcare access based on gender. South Africa has shown commitment to resolving gender imbalances in healthcare, historically emphasizing cisgender women’s challenges. However, research gaps exist in exploring how public health systems perpetuate disparities among gender-diverse persons, like trans women, who face exclusion due to their deviation from cisgender norms in healthcare. Critical, intersectionality-informed health research carries the potential to reveal the diversity of gendered healthcare experiences and expose the systems and processes that marginalize trans patients.

**Methods:**

This study adopts a critical trans politics perspective to explore the socio-political forces limiting South African trans women's access to public healthcare. Using a critical narrative approach, the research asks:

1) What narratives do South African trans women share about their experiences in health systems?

2) What gendered societal structures, practices, and norms enable or hinder their inclusion in health systems?

Over a period of two months in 2022, five South African adult trans women between the ages of 22 and 30 participated in 60 to 90-min long, semi-structured individual, telephonic interviews, focusing on participants' subjective experiences in healthcare.

**Results:**

Trans women's narratives unveiled a culture of medical genderism in South African public healthcare, discriminating against patients whose gender misaligns with societal norms. This culture is represented by the trans women's experiences of their identities being structurally stigmatized and delegitimized when seeking healthcare, reflected in institutional policies, practices, and protocols consistently disregarding and misgendering them. Trans women’s systemic erasure was illustrated by the restricted professional knowledge, availability, and adoption of gender-affirming healthcare in a ciscentric public healthcare system prioritizing cisgender needs. The intersection of gender, race, and class dynamics compounded the obstacles faced in accessing healthcare.

**Conclusions:**

This inquiry underscores the structural hurdles trans women face when accessing suitable public healthcare. It introduces a gender equity framework for trans inclusive healthcare, outlining implications for research, theory, policy, and practice. Toward the goal of embracing complexity and diversity, this framework, for example, promotes the rigorous absorption of trans persons and their healthcare experiences in gender-responsive programming, and encourages the development of a comprehensive understanding of gender equity from an intersectional perspective incorporating the unique needs and rights of trans healthcare seekers. The framework also offers practical guidance for cultivating health systems attuned to gender diversity (such as addressing medical genderism and recognizing the broad spectrum of identity at a policy level).

## Background

Globally, public health research and policy reform addressing healthcare disparities has for the most part neglected to examine the structural mechanisms that maintain the exclusion of trans patients in health systems. According to Gilson [13], health systems, as an extension of society, do not only produce health and healthcare but they are also purveyors of a wider set of societal norms and values. As such, these systems are molded by broader socio-political contexts [17] and biases that inform the institutionalization of care [6]. These biases create considerable barriers to equitable healthcare access [15], reinforcing pathways to conditions of societal exclusion [18], especially for minority groups. In a global milieu marked by the continuing social exclusion of women in various socio-geographic settings, imbalances of power in health systems along the lines of gender and sex have been theorized to systemically perpetuate disparities in healthcare for women [42]. Recently, sexually and gender-diverse persons are being theorized to carry the burden of contending with biases brought up by complex systems of power in medicine that place cisgender heterosexual persons at the top of the social hierarchy. Such gender power systems afford privileges to cisgender patients while disadvantaging patients who reject the norms of the gender binary [11, 43], within a larger healthcare system that is organized from a cisnormative framework that centers on the experiences, needs, and rights of cisgender patients [3, 43]. Trans women navigate nuanced layers of exclusion in healthcare as they carry what Jeane and Janes ([22]: p. 1238) describe as “the double bind” of being women and trans, navigating health systems as women in patriarchal medical worlds [18, 37] and as queer and trans in cisnormative health systems [1, 29].

One of the largest trans discrimination studies conducted, with a sample of 6 450 trans study participants based in the United States of America, found that 50% of the sample reported having to teach their medical providers about trans-affirming care [16]. Of the total sample, 48% reported an inability to afford healthcare and 28% reported postponing medical care due to discrimination [16]. Studies conducted in contexts such as Canada [14], Australia, and New Zealand [41], as well as South Africa [50], reveal high incidents of anti-trans stigma in healthcare settings. The studies show that being trans in health systems remains a facilitator of unfair and unavoidable differences, supporting the narrative that health systems are certainly not gender-free.

Intersectionality is an innovative gender analytical framework developed by Crenshaw [7] that speaks to the overlapping nature of structural oppression. When addressing gender inequities from a public health perspective, an intersectionality-informed approach holds considerable significance in recognizing the varied spectrum of identity experiences in health systems and carries the potential for making trans women visible in public health research [3, 28], Intersectionality is particularly important for examining the specificities of the social location of trans women in public healthcare systems, enabling the development of new trans-centric theoretical concepts that allow scholars and practitioners to understand the structural determinants that engender unique forms of oppression for trans women in healthcare [2]. A more thoughtful incorporation of intersectionality can promote meaningful interventions, such as policy changes, that are specific enough to the population under study [2].

This study focuses on trans women’s experiences within public healthcare systems in the post-apartheid South African context, a nation that remains deeply divided in terms of gender, race, and space [40]. Public health research on trans experiences in South African healthcare is slowly shedding light on historically marginalized gendered experiences. Campbell and colleagues [5] report that 82% (*n* = 57) of South African transgender adults receiving gender-affirming healthcare at the Steve Biko Academic Hospital Gender Reassignment Clinic in Gauteng and the Groote Schuur Transgender Clinic in the Western Cape indicated experiences of social exclusion due to societal stigmatization of their gender identity, predominantly from family members, with these experiences revealed to be significantly correlated with psychological distress. Studies such as by Muller [35] based on trans participants living in the wider Cape Town area in the Western Cape province (one of the provinces with the greatest health resources) as well as in the urban and peri-urban areas near Johannesburg in the Gauteng province have found that trans individuals face prejudiced treatment and discrimination across various South African healthcare settings. This discrimination has been established to be rooted in a lack of acknowledgment of trans people as patients within the health system [31]. Luvuno and colleagues [31] in their study covering rural, urban and peri-urban settings in the KwaZulu-Natal province reported that patients who are trans are commonly met with poor prejudicial reception in healthcare institutions. Mbeda and colleagues [33], exploring the factors associated with healthcare-related stigma in Kenya, Malawi as well as in Cape Town and Soweto within South Africa suggest that, as a result of this prejudice, trans persons are afraid to seek healthcare services. Trans women’s exclusion in healthcare is discovered to be further aggravated by a lack of skilled healthcare workers, policies, and programs addressing the needs of trans patients [31].

Validating the absence of policy and policy implementation, Spencer and colleagues [46], in a South African study representing healthcare providers from Gauteng, Western Cape and KwaZulu-Natal, argue that the South African government has not formalized gender-affirming public health systems that can safely facilitate the medical transition of trans persons. Only six South African national government hospitals currently provide gender-affirming care, with waiting lists for gender-affirming surgeries of up to twenty-five years [46]. Gender-affirming public health systems and specialized trans units are limited, with only one hospital in the Western Cape currently offering a dedicated Transgender Unit [51], severely limiting trans persons’ access, especially those dependent on state resources. In fact, the majority of the public, up to 71.5% of the entire population [47], relies on an underfunded and poorly managed public healthcare system in South Africa [32]. This statistic emphasizes how important public healthcare services are in South Africa and how critical it is that they remain functional, sensitive, and fairly accessible to the diverse range of health users accessing the system, including trans women.

This study employs a critical trans politics (CTP) perspective to examine the socio-cultural and political dynamics that restrict trans women’s access to public healthcare. CTP seeks to deconstruct and challenge oppressive structures affecting the lives of trans individuals [45]. The study focuses on gender as an institutional construct manifested in public healthcare policies, practices, and entrenched cultural norms that disadvantage trans women. Concepts such as genderism and cisnormativity are central to understanding and addressing trans marginalization in health systems. Genderism understands gender as a fixed binary composed of man and woman [25], while cisnormativity conceptualizes gender as a binary category based on one’s sex assigned at birth [12]. By adopting a critical approach, the study aims to explore the lived experiences of 5 South African trans women and in so doing uncover institutional barriers to access to healthcare in local public service settings.

## Methods

This research draws on a critical narrative approach [10], using storytelling as a meaning-making tool to facilitate a better understanding of South African trans women’s experiences in health systems. The study aims to understand:

- 1) What narratives do trans women living in South Africa tell about their experiences in the context of public health systems?
- 2) What are the gendered societal structures, practices, and norms that enable and facilitate the inclusion and exclusion of trans women in South African public health systems?

A critical inquiry explores how individual stories are mediated by broader sociocultural and political contexts, explicitly deconstructing stories to address and question the construction of knowledge, power, and reality entrenched in and through narrative accounts of human experience [21, 36].

### Participants

Five South African adult trans women participated in this study, ranging in age from 22–30 years. Trans women are defined here as women whose self-defined gender identity does not align with the gender that was socially assigned to them at birth [49]. While the sample size was relatively small, the study focus was on capturing the social context that shapes trans lives, eliciting deep, detailed accounts of participant experiences and the sample met this purpose. Trans women in this study are defined as women whose self-defined gender identity does not align with the gender that was socially assigned to them at birth [48]. Participants represented Gauteng, Limpopo and the Northern Cape provinces of South Africa. Despite the economic inequalities that persist, Gauteng is a relatively large, urban province with strong infrastructure, economic opportunity, and cultural diversity, while Limpopo and the North West provinces are more rural, less infrastructurally developed socio-geographic contexts. All participants had engaged with public healthcare facilities. Some participants were employed, while others were looking for employment. To safeguard the privacy of participants, pseudo-names were used when reporting research findings.

### Sampling strategy

The study used a purposive sampling technique. The first author (SS) created a digital poster and published it on their social media platforms – namely, Facebook, Instagram, and WhatsApp. Their social media community was requested to share the posters widely. Interested participants contacted SS directly and none of those who reached out to participate in the study later withdrew consent. None of the participants had an existing relationship with either author.

### Data collection

Semi-structured individual, telephonic interviews, 60 to 90 min in length, were conducted over a two month period in 2022 where questions regarding participants’ subjective experiences within the healthcare sector were explored. To facilitate exploration, the following topical questions were asked: “How has being a trans women impacted your healthcare experiences? What difficulties do you typically experience when accessing healthcare services? What has been positive about your experience in healthcare settings?” The telephonic voice calls were recorded via a Microsoft PowerPoint recording function. The data were transcribed verbatim for analysis.

### Data analysis

Fraser’s [10] phased analytical procedure was used to analyse the data from a critical narrative perspective. The two authors (SS and MC), working independently, immersed themselves in each transcript, noting emerging themes and types of stories. To avoid a narrow perspective, the researchers explored the experiences across different dimensions, including intrapersonal, interpersonal, cultural, and structural aspects. They connected the themes to larger societal structures and systems of power, considering factors such as class, gender, race, sexual orientation, age, (dis)ability, religion, and socio-geographical location. The researchers then shared and compared their analyses. SS then contrasted and reflected on the content, type, and tone of the narratives, and translated the narrative content into a formal written analysis.

## Results

The five participants in the study were not only navigating public healthcare systems as women, but they also happened to be black and socioeconomically positioned as poor and working class. They were dependent on state resources, with their stories representing predominantly public healthcare experiences across different provinces in South Africa (see Table 1).

**Table 1:** The demographics of the participants who were sampled and interviewed in this study

Pseudonym	Age	Preferred pronouns	Gender	Province
Phumeza	29	She/her	Trans woman	Gauteng
Sam	30	She/her	Trans woman	Limpopo
Lerato	29	She/her	Trans woman	Limpopo
Laila	24	She/her	Trans woman	Northern Cape
Mpho	22	She/her	Trans woman	Gauteng

Three dominant thematic narratives emerged, which broadly spoke to the positioning of trans women as stigmatized others in ciscentric public health systems that maintain and reinforce traditional norms of the gender binary. This positioning favours cisgender positions within public healthcare while invisibilizing and excluding trans positions. The first theme highlights the denial of trans women’s self-determination reflected in institutional policies and practices that misgender trans women, limiting their access to dignified care. The second speaks to the lack of knowledge about gender-affirming healthcare held by healthcare professionals and healthcare institutions at large and played out through trans-erasure. The third speaks to the barriers to accessible and equitable healthcare faced by trans women compounded by the collusion of gender, race, and class structural dynamics.

Structural (mis)gendering and the denial of trans women’s self-determination: “If they respected me, they would have put me in a female ward.”

Uncovered by the participants’ stories is the institutional refusal of their right to access health systems as women, as their self-determined gender, where policies, procedures and practices in public healthcare consistently fail to recognize and respect their trans identities. This is demonstrated through the limited accommodations that are made for their needs and positions as trans patients in cisnormative public healthcare settings that operate on the assumption that every patient is, and should be, cisgender. For example, Lerato said: *“If you have to go to the clinic, you will be judged. Sometimes you will be judged by the security at the gate for your gender, how you look and stuff. So, you end up not going to the clinic”*. Sam shared a similar experience: *“Yes, you will feel excluded the moment you enter the gate. They will make funny jokes about your sexuality, which is totally wrong”.* In the same way, Laila conveyed a story of being received with a negative attitude by an administrator when reaching out for healthcare: *“They told me… it’s going to be very expensive, so I must go to a local clinic… And then the clerk there [at the local clinic], she gave me attitude.”* All these stories reflect how trans women in this study felted judged and made fun of for how they looked and chose to express their gender. Participants experienced themselves as unwelcomed at multiple levels in healthcare settings which speaks to a stigmatizing culture within these systems.

These gendered societal attitudes and perceptions are driven by macro-level societal discourses about transness that create powerful barriers to accessing healthcare. For example, Sam highlights how her gender identity needed to be confirmed as soon as she entered the gates of the hospital: *“There is security, right? By confirming your identity, they will see that you are a man”.* This practice of confirming identity, especially within sex-segregated medical settings, has important and serious consequences for a woman like Sam, whose gender self-identification does not mirror the gender category she was medically and legally accorded at birth. This is a practice that constrains Sam’s opportunity to receive medical care as an already stigmatized member of society. After the confirmation of Sam’s identity upon admission for the treatment of tuberculosis, Sam was allocated to a male medical ward as per her medico-legal sex designation at birth: *“I stayed at the hospital for 2 weeks for treatment due to TB, living with males in the same ward… Remember it’s a public hospital. I didn’t even have the power to ask for a single room or a single ward.”*

Cisnormativity, the assumption that every person has and should have a gender that assigns with the gender they were assigned at birth [1], and gender fundamentalism, the view that entrenches gender as a fixed, innate and dichotomous category [6], become the ideological premise of Sam’s experience of being misgendered in the public hospital, where she is addressed and treated using language and designations that do not reflect the gender with which she identifies. In a sex-segregated medical system, stringently divided along rigid gender lines, Sam’s allocation to a male ward demonstrates the perpetuation of an enduring custom of assigning gender and imposing identity in a way that denies individual agency and restricts human rights. Sam specifically mentioned that she did not even have the power to voice her wishes, speaking to a position of social subordination she held in relation to cisgender medical power that held the authority to designate gender.

Mpho had a similar experience, where her gender self-identification was disregarded as a psychiatric in-patient: *“I was not staying in the female or male’s room. I was staying in between the female rooms and male wards… Oh, it was so painful. I didn’t feel like I was respected much, that my privacy was respected… Because if they respected me, they would have allowed me to go to the female bathroom and put me in a female ward but they didn’t do that.”* In sex-segregated systems such as the hospitals that Mpho and Sam found themselves in, they were required to choose between enduring the indignity of accessing services according to their natal gender or gender assigned at birth and foregoing services entirely. Through these narrative accounts, the South African public healthcare system is represented as a social structure set up in ways that assume cissexuality, and in so doing negates patients’ rights to self-identify.

Although the majority of experiences in this study highlight misgendering and consequently stigmatization in pathologizing public healthcare settings, there were the occasional experiences of inclusion. For example, Mpho described the power of simple experiences, such as being addressed and treated by medical staff in ways that affirmed her identity: “*Some people were not [supportive]. They were, like, “Huh? And then? Some people respected my choice, and they respected everything. They respected me, and they started calling me using feminine pronouns, and I really appreciate that because that really helped with my transition”.* Phumeza also shared experiences that constituted being addressed and treated by the people around her in ways that affirmed her identity: *“I’ve worked for the company and now I am the patient there, however, the treatment is still the same and I am happy that when I go there, I am actually me and I know what to say there.”* These experiences, while infrequent, provide a glimpse of what inclusive healthcare may look like.

The knowledge gap and institutional silence on gender-affirming healthcare: “You encounter certain staff members that have no idea about being trans.”

Participants’ narratives speak to how healthcare professionals and public healthcare institutions largely lack knowledge about gender-affirming healthcare. Trans erasure, whereby trans patients' needs are neglected due to the lack of trans-inclusive policies and practices in healthcare structures [1] was evident in these participants’ experiences. This erasure is a powerful consequence of the failure of knowledge systems to respect, safeguard and acknowledge trans identities, rendering these patients invisible within the public health system. Sam shared the following experience: *“Let me make an example, if you go to a public health facility, you will find there is someone who doesn’t even know about the transition. So, it starts with the health facilities, the public health around Limpopo is very, very cruel… You encounter certain staff members that have no idea about trans. They don’t have any idea about MSM”.* Laila shared a similar narrative of reaching out for gender-affirming hormone therapy and finding out that the healthcare staff was not aware of gender medical transitioning as a legitimate medical procedure: *“So, I went to the local clinic and then they didn’t have a clue what it [transitioning] was all about. And then the clerk there, she gave me attitude”.*

Both Laila and Sam struggled to find a trans-inclusive public healthcare environment, where medical and support staff demonstrated specific knowledge of trans health issues. These two experiences of the study participants speak to the denial of their existence as trans patients within public healthcare systems. This denial has a direct connection to the denial of their healthcare needs. Laila further reports: *“They don’t know about it; they don’t keep it [hormone treatment]. They don’t know how to help you start with the process; whom to refer you to, where you go, how to start and whom to help you”.* Laila’s and Sam’s engagement with public healthcare suggests that (even though the information may be present) this information may rarely be incorporated into official healthcare protocols and processes, nor integrated into the educational training of healthcare practitioners in South Africa.

Mpho challenges the South African public health system’s lack of provision of gender-affirmative surgeries, suggesting that the public health system does not seem to prioritise their needs as trans identities: *“They don’t want to perform surgery on us because they are constantly telling us that they are currently performing more urgent surgeries. They are performing for cancer patients and all that. I don’t know why. It’s an excuse not to help us. I see it as an excuse”.* Mpho questions the extent to which public healthcare takes seriously the health needs of trans persons. This is a reasonable charge in light of historical cisnormative practices in medicine that have pathologized trans populations [26]. This unavailability of resources possibly speaks to the lack of integration of gender affirmative care into mainstream public healthcare. Particularly, it spotlights the participants’ marginalisation in public medical practice.

Lerato contends that the underrepresentation of gender-diverse healthcare practitioners is also key to their social exclusion in medicine: *“I mean, if you are a trans, you should be able to see that this is a trans person. If at the clinic we meet trans women, there should be a trans woman who will deal with the health issues… If there was a trans person at the clinic who would accommodate someone like a trans person, you would feel safer because we are the most vulnerable ones”.* Lerato urges for increased representation and visibility of trans healthcare providers. The essence of Lerato’s petition is a concern about the incorporation of trans-inclusive practices and protocols in medical settings that make it easier for trans patients like herself to seek care, self-identify, and have their healthcare needs competently met.

The importance and value of Lerato’s plea is reinforced by Phumeza’s positive experience of a trans-inclusive public clinic in her community: *“Actually, when I go to the clinic, they just talk to me like a woman. They even say to me if I’m not feeling well. They would say to me [that] if I want to go to the toilet, they can they come with me and all those things. So, I actually, just laugh about the whole situation. I’m glad that we have our own choice clinic… I get my hormonal treatment there and any other clinical services. So, if I have flu, your Gonorrhea or let’s say that any STI/STD of some sort, they are able to help me with that.”* Lerato’s and Phumeza’s reflections provide examples of how inclusion in healthcare may look and feel for trans women in South Africa. They emphasise the importance of institutionalising public healthcare practices that celebrate gender diversity of healthcare providers that are educated on trans health, administration staff that is informed about how to communicate appropriately with trans patients, and medical infrastructures that are organised in such a way that trans patients are not imposed gender assignments that do not mirror who they are.

(In)access to trans-specific healthcare services – a race/class/gender intersection: “I wanted to start with my hormone therapy but then I couldn’t because there was the issue of money.”

Participants in the study are not only navigating public health systems as women. They also happen to be black and socioeconomically positioned as poor, navigating a post-apartheid healthcare context pervaded with historical racial, class, and gender inequalities. These narratives highlight how primary public medical institutions do not have sufficient resources to provide gender-affirmative care for trans patients. Laila speaks of the inaccessibility of hormonal replacement therapy due to the severe resource limitation of state-funded public clinics: *“The only thing is that the resources on this side. It is like we do not have resources. You will go to the clinic and you will hear that they don’t have enough estrogen. It’s not easy at all because sometimes you might have to go and buy yourself with your own money when there are no resources. There are even clinics in the Northern Cape that don’t even keep them in their clinic”.*

Due to this lack of resources, the desperation to medically transition and self-actualise pushes women like Lerato to explore alternatives, which are often unsafe. Lerato obtained gender-affirmative treatment through illegal trade without medical supervision: *“For a trans woman like me, it’s a lot of needs because I have to go for hormone pills. And I am taking them from the black market because it’s very expensive to consult the doctor and that… So, in Limpopo, you would go for birth control pills at the [public] clinic. And because I have a lot of friends who are [cisgender] girls, they would give me Triphasil pills and they help you develop some boobs. But those ones I was on them, there were a lot of complications. The last time I was on them, my male part wasn’t working properly and I had some terrible cramps. I had to drop them then this other friend said I should go for hormone pills”.*

Lerato’s use of professionally unapproved medication led to frightful health complications, which included cramps and the malfunctioning of her penis. Lerato, thus, addresses her inaccessibility to professional and safe healthcare as manifestly driven by the unavailability of resourced public healthcare facilities and the unreliability of public benefit organisations that provide healthcare services often for only a short term: *“It’s very difficult for us. We are on a low scale, a very bad scale… You would find that certain organisations would come and provide lubricants and stuff like that. So, after 6 months that organisation is no longer there, and contracts are terminated because there is no longer funding or something like that. So, I would have to go back to my normal… It’s very tiring because we no longer trust anything. So, that’s why I’m saying we’re on a low scale. You go and consult for a few months but after that, you don’t have money so you see it’s a long process”.*

Participant narratives highlight experiences of being unable to afford the gender-affirming medical care they needed. The industrialized private medical sector in South Africa is seen in these examples to construct healthcare practitioners as healthcare providers while patients are constructed as consumers. Lerato’s and Sam’s narratives capture the process of seeking medical care as an economic procedure that requires financial capital they often do not have. Lerato explains: *“You go and consult for a few months but after that, you don’t have money. So, you see, it’s a long process”.* Sam shares similar experiences: *“I only worked for only 2 years and 6 months and my contract has ended due to COVID. So I was home, I couldn’t even go for laser therapy, I couldn’t even go, the moment I became broke, I became broke. I wanted to start as soon as possible with my hormone therapy but then I couldn’t because there was the issue of money”.* Due to this commodification of healthcare, economically vulnerable trans women are excluded from meeting their healthcare needs.

To the problem of racialised and classed healthcare inaccessibility, trans women like Lerato and Sam, consequently, often find themselves having to travel long distances to access services in the few public hospitals in the country where the government has instituted gender affirmative care. Sam finds that she has to travel across provinces to access gender-affirming care: *“You need to go to the Gauteng Province; you need to go to Cape Town. The hormones are very insufficient. You need to go to other provinces to get medical resources”.* Space, as a legacy of apartheid segregation, in the narratives of the participants is mirrored as reflecting and reinforcing socioeconomic inequality. Lerato, who lives in rural Limpopo, has to travel to Johannesburg (a journey of almost 400 km) to access gender affirmative care: *“There is no doctor in Limpopo, you have to go to Wits. I had to go to Baragwanath Hospital and I was put on a waiting list till today… Yes, for the consultation, for the psychologist, everything”.*

The economic vulnerability of women like Lerato, who reside in rural communities situated at the socio-geographical and economic margins of society, makes their reach to medical institutions challenging due to the travel costs involved. Due to her limited financial capabilities, Phumeza highlights that she has needed to prioritise her basic necessities, which sometimes comes down to a choice between food and transport costs for healthcare services: *“Sometimes I struggle to go to the clinic because I only have one source of income and I have to make sure that it lasts me let me say probably the whole month because I have to buy food, toiletries and everything you know”.*

## Discussion

The narratives shared by the South African trans women in this study show how gender is not solely based on assumed natural and fixed categories but that gender is socially constructed and regulated, even within healthcare systems. The participants' stories bring attention to a dominant structure of genderism entrenched in South African medical institutions, whereby genderism is manifest as an ideological framework that “reinforces the negative evaluation of gender nonconformity or incongruence between sex and gender” ([20], p. 534). Sampson ([44], p. 35) defines this framework of genderism as constituting “the belief that gender is binary, and that only two genders – male and female – exist.” Within the context of a medical system shaped by this gender structure, trans women’s participation in the South African public healthcare system is seen to be constricted by biases and norms surrounding an institutionalized gender binary that does not account for fluidity, difference, and the broad spectrum of gender identity and expression. Within South African public healthcare settings, trans women are discovered to be faced with what can be conceptualized as a structural framework of medical genderism – managing the administration and provision of healthcare from an underlying ideological investment in a binary gender model and the imperative for alignment between a healthcare user's gender and their birth-assigned sex. This structure of medical genderism centers, prioritizes and caters to the cisgender healthcare user, who conforms to established norms of the gender binary in medical settings, while neglecting, discriminating against and/or denying the existence of the gender-diverse healthcare user, who experiences pathologized incongruence between their self-defined gender and societal norms associated with their birth sex.

From participants' narratives, medical genderism was evident through the pervasive interpersonal stigma perpetrated by cisgender staff members against trans healthcare seekers. This stigma appears to be driven by power dynamics and broader societal discourses present in public health systems that continue to discursively construct trans individuals as pathological and socially deviant. In this study, medical genderism was revealed to underpin and strengthen a phenomenon of institutional erasure. This phenomenon, speaking to systems that structurally fail to accommodate trans identities [1, 30], was echoed by participants’ experiences of their identities and healthcare needs being systemically neglected in ciscentric public healthcare facilities that center a cisgender worldview and lack policies, protocols, practices, and customs that are inclusive of gender-diverse patients. Within such a ciscentric medical culture, institutional erasure is reinforced through chronic misgendering perpetuated by healthcare professionals’ manner of address, along with supporting staff, invalidating, ignoring, and undermining the self-determined gender identities of trans patients. Institutional erasure is further strengthened through the implementation of sex-based healthcare segregation. Public healthcare systems systematically impose the placement of patients into rigid gender categorizations on the basis of one’s birth sex and administratively render healthcare on the premise of these binary categorizations. This gendering of public healthcare appears to be driven by an ideology of gender essentialism, historically deeply rooted within the field of medicine. Under this ideology, gender is systematically reproduced and enforced as a social structure composed of immutable, inherent, and biologically determined binary categories of man and woman [4, 6]. As a result, the trans women in this study (assigned ‘male’ at birth and gendered as ‘boys’) detailed stories of being forcefully allocated to ‘male’ wards despite self-identifying as women. Trans individuals are revealed to be constantly followed and burdened by sex-based labels of gender identity imposed on them by cisgender medical systems (alongside cultural and legal systems) possessing the authority to designate gender, from the time they enter the world.

Within the contextual backdrop of medical genderism, the participants’ healthcare experiences of being structurally disregarded also shed light on an underlying blueprint of cisnormativity [27] apparent in a public health system that assumes all patients have – and should have – a gender that corresponds with their assigned sex at birth. Cisnormativity is perpetuated through the delivery and organization of a public healthcare system that normatively centers on patients whose gender self-definition corresponds with birth sex and assumes their needs and identity experiences as the standard, while, as Lampe [29] noted in their study, compelling trans patients’ adherence to these limiting norms when accessing healthcare. In a Canadian study by Bauer and colleagues [1], this is exemplified through medical data and protocols for testing or treatment that are gender-specific and assume all patients are cisgender, or intake forms that do not allow for trans patients to self-identify as their self-defined gender. Similarly driven by cisnormative processes, public healthcare facilities in South Africa are recognized as coming up short when it comes to achieving gender-based equity, failing to implement systems that accommodate and respond sensitively to the gender-diverse healthcare seeker.

The trans women’s experiences in a cisnormative healthcare system also emphasized the problem of the informational erasure of trans patients in South African public healthcare. Informational erasure, a concept initially outlined by Bauer and colleagues [1], describes the side-lining and repression of information related to trans issues, experiences, and identities in healthcare,perpetuating ignorance about trans patients and maintaining their systemic neglect and invisibility. Speaking to this, the invisibilisation of gender-affirming healthcare services in mainstream public healthcare emerged as a dominant narrative in this study. Brought into focus by the participants were public healthcare facilities that were unable to demonstrate adequate medical knowledge pertaining to trans-specific healthcare needs, from executives managing the facilities to the treating healthcare professionals. This problem extends itself as an epistemological function of medical genderism, speaking to how processes pertaining to the creation and distribution of medical knowledge are coherently guided by gender biases that undermine and discriminate against trans individuals.

At the heart, medical genderism, central to both the institutional and informational erasure of trans patients in public healthcare, challenges the trans patient’s ethical right to health. It undermines diversity and adds to significant healthcare disparities for trans individuals compared to cisgender individuals. These disparities have been demonstrated by previous studies from Giblon and Baur [14] in Canada as well as by Fieldman and colleagues [9] in the United States, for example. Alongside studies by Luvuno and colleagues [31] together with Mbeda and colleagues [33] in South Africa, similar to this study, a spectrum of studies has especially drawn attention to the healthcare exclusion experiences and biases trans populations face in the Southeast of the United States [24, 29], New Zealand [39], United Kingdom [19], India as well as Pakistan [34, 38]. These findings reflect the pervasiveness of the exclusion of gender-diverse persons in healthcare settings in various parts of the world. However, despite the pervasive challenges faced by trans individuals with accessing equitable healthcare, some positive experiences of acceptance, gender diversity, and inclusion in a few medical cultures were reported by the trans women in this study. These often subtle reported experiences of inclusion revealed cisgender institutional power as not immune to resistance and transformation.

The narratives of the participants, predominantly black, poor, and working-class trans women navigating a post-apartheid South Africa context, also highlight the intersecting factors of race, class, and gender that create compounded barriers to healthcare access. Commodified gender-affirming treatment in a well-resourced industrialized private healthcare system was experienced by the women as grossly unaffordable and inaccessible. Gender-affirming medical treatment had limited availability in profoundly under-resourced and poorly managed state-funded medical facilities [23], challenging public healthcare access for the trans women, economically dependent on state services due to their socio-economic status. Due to the lack of gender-affirming medical services, especially at the regional and district public healthcare facilities [23] closer to where most participants live, the economically disadvantaged trans healthcare seeker, located in the rural and township socio-geographic outskirts, found themselves unable to meet the costs of traveling long distances to the few academic hospitals (mostly located in major city centres) that have instituted gender-affirmative care. Therefore, due to racialized economic inequalities and the capitalist commodification of both mobility and health in post-apartheid South Africa, in interaction with the problem of medical genderism in a public healthcare system that neglects trans patients, the marginalization of the study participants was deepened, further complicating healthcare access.

## Limitations

Although the use of a five-person sample size is arguably appropriate for an exploratory and critical inquiry, the study findings do not map a full picture of the lived experiences of trans women in South Africa. Instead, the study offers an important glimpse at normative ideologies, practices and systems that maintain the societal marginalization of trans women in post-apartheid South African healthcare. The perspectives of trans men and gender nonconforming persons are missing from this account. However, in line with trans* epistemology, this study embraces the diversity and plurality of trans subjectivity, recognizing that there is no singular trans experience.

## Conclusions

This study is significant for its innovative approach in shedding light on the contextual influences of medical genderism, a binary structure of gender that perpetuates the neglect of gender-diverse patients in post-apartheid South African public healthcare. Trans women's neglect in medicine and their challenges with accessing gender-sensitive healthcare overall arise from existing outside the bounds of entrenched ciscentric genderist structures in which care is regulated from a position that does not have trans patients in mind and, thus, challenges the gender-diverse patient’s ability to receive healthcare that meets them where they are in terms of their needs. By amplifying the stories and experiences of gender-diverse healthcare seekers and users, historically situated at the gender margins in medicine, this study empowers the voices of those who are marginalized in healthcare. It provides a platform for a discursive reimagining of the social world and specifically public healthcare from a historically silenced trans standpoint. There is an urgent need to move public healthcare systems in South Africa beyond the gender binary towards a gender equity framework that not only recognizes gender diversity but celebrates it.

In foregrounding the unique experiences of gender-diverse persons in healthcare, compelling concerns about the inequitable arrangements of public healthcare in South Africa are raised. In working against trans marginalization in public health systems, a gender equity framework for trans inclusive healthcare is proposed to inform theory, research, practice, and policy. Such a framework is important for shaping the revision of how healthcare is currently delivered and managed in South Africa, moving health systems from rigid and limiting norms of the gender binary toward equity-based reform that embraces the gender diversity of healthcare users.

Conceptually, a gender equity framework for trans inclusive healthcare advocates for:

i. The rigorous inclusion and absorption of trans persons in gender programming, especially in light of their historical invisibilisation in public health.
ii. A greater refinement of the concept of gender equity from an intersectional perspective – recognizing the heterogeneity of gender-based experiences in public healthcare and addressing the diverse barriers to care shaped by cis-hetero-patriarchal gender hegemonies that position the needs of those cisgender, heterosexual, and male as superior in health systems.
iii. The mobilization of medical genderism, a theory of genderism in the context of healthcare, in the interest of naming, marking and interjecting medical legalities, administrative processes, policies, discourses, practices, customs, and norms that are gendered in ways that exclude trans persons in healthcare structures – always reproducing trans healthcare users as pathologically non-normative and out of the ordinary, while centring and prioritizing cisgender healthcare users.
iv. The adoption of critical trans politics as a socio-political perspective contributing to assessing and addressing trans patients’ (in)access to healthcare from a structural viewpoint. This critical perspective carries the potential to render trans lives politically visible in national and global public health initiatives challenging healthcare disparities – giving priority to mapping and challenging administrative processes and structures of power in medicine that specifically oppress gender-diverse healthcare users.

When it comes to policy and research:

i. The framework argues for taking medical genderism as a social justice issue and a serious human rights concern in public health.
ii. In order to transform the current logic of state healthcare, gender-transformative approaches need to be explicit and intentional in including diverse gender identities in policies, practices, organizational cultures, and data.
iii. A move toward discourse analysis and critical policy ethnography as a central piece in health research for trans patients is supported. This is so to enable a nuanced ground-level analysis of the discourses and policies [8] in medicine that maintain medical genderism, sustaining the exclusion of trans persons in South African healthcare. Discourse analysis and critical policy ethnography shall empower the possibilities for medical gender structures to be clearly viewed, deconstructed, and interjected,opening up opportunities for inclusive gender reform that solves the practical problems of trans persons in medicine.
iv. In pursuit of democracy and fostering a constitutionally just society, the South African government holds a duty toward allocating adequate investments to research and programming that makes trans patients and their needs heard and seen in public health discussions – interrogating the elements of the existing medical paradigm that do not adhere to the values of democracy, equality and ubuntu.

When it comes to practice, the framework posits that institutionally countering the systematic erasure of trans persons in public healthcare contexts is in and of itself, a crucial step toward providing an intervention that supports the health and well-being of trans persons in society. As Bauer and colleagues [1] have put forward before, there is a need for more resources to be made available to professionally guide attending to the healthcare needs of trans persons. The following are possible recommendations for inclusion, inspired by those penned by Bauer et al. [1]:

i. Developing intake forms that allow trans patients to self-identify. Through these means, the administration is handled in such a way that it does not restrict self-identification and self-determination.
ii. All healthcare providers and supporting staff should use pronouns and names appropriate for a patient’s gender identity.
iii. For healthcare practitioners to assume that any patient may be trans. In other words, for healthcare practitioners to divorce themselves from the limited assumption that every patient is cisgender.
iv. Developing protocols for assessing, diagnosing, and treating that are not gender-specific or that do not assume all persons are cisgender.
v. All in all, eliminating sex segregation where possible and providing a safe place for trans patients to access care without entailing a contravention of their ethical right to self-determination in healthcare structures.

## Data Availability

The datasets used and/or analysed during the current study are available from the corresponding author upon reasonable request.
